# Modulating Brain Oscillations to Drive Brain Function

**DOI:** 10.1371/journal.pbio.1002032

**Published:** 2014-12-30

**Authors:** Gregor Thut

**Affiliations:** Institute of Neuroscience and Psychology, University of Glasgow, Glasgow, United Kingdom

## Abstract

Do neuronal oscillations play a causal role in brain function? In a study in this issue of *PLOS Biology*, Helfrich and colleagues address this long-standing question by attempting to drive brain oscillations using transcranial electrical current stimulation. Remarkably, they were able to manipulate visual perception by forcing brain oscillations of the left and right visual hemispheres into synchrony using oscillatory currents over both hemispheres. Under this condition, human observers more often perceived an inherently ambiguous visual stimulus in one of its perceptual instantiations. These findings shed light on the mechanisms underlying neuronal computation. They show that it is the neuronal oscillations that drive the visual experience, not the experience driving the oscillations. And they indicate that synchronized oscillatory activity groups brain areas into functional networks. This points to new ways for controlled experimental and possibly also clinical interventions for the study and modulation of brain oscillations and associated functions.

## Abstract 2

In this transcranial alternating current stimulation study, Randolph Helfrich, Andreas Engel and colleagues demonstrate that the selective modulation of synchronized neuronal activity in large-scale cortical networks impacts conscious perception.

How does the brain work? How does it code, transfer, and store information? How are conscious experiences generated? These, among others, are long-standing questions neuroscientists try to answer. One way to approach this is to study how the brain orchestrates behaviour, for instance, by measuring brain activity and relating it to behaviour. Yet, studying the brain–behaviour relationship raises another series of questions: What type of brain activity should one look at? Do we need to record directly from single neurons? Or can we make inferences also by recording from larger pools of neurons? And importantly, do these measures of brain activity provide mechanistic accounts of how the brain implements function, or are they just inevitable side-products, with limited explanatory power for the neural mechanisms underlying our experiences, thoughts, or actions?

Certainly, one would have a good argument for brain activity causally underlying brain function if (i) this brain activity not only relates to sensory experiences or behavioural performance measures (revealing a correlative brain-behaviour relationship), but (ii) interventions into this brain activity would also modulate our experiences or performance (revealing a causal link). Recent developments allow addressing these central points for oscillatory brain activity, which is what Helfrich et al. [1] did in their study published in this issue of *PLOS Biology*.

At the basis of Helfrich et al.'s study are two lines of research, one of which is concerned with the interpretation of a special type of brain activity, namely, brain oscillations. This type of brain activity represents voltage fluctuations of neuronal elements and was initially observed from one scalp electrode by Hans Berger [2]. Today, brain oscillations are typically recorded from multiple sensors distributed over the scalp or brain, for instance using electro- or magneto-encephalography (EEG/MEG), in order to make inferences about the orchestration of brain activity across distinct neuronal elements [3]. A prominent view is that these oscillations represent essential network activity. They become visible when neuronal elements of a network start to synchronize their oscillatory activity, i.e., temporarily couple together [4]. Notably, brain oscillations vary in frequencies depending on the task that is being executed and the region of the brain they are recorded from [3] (see Box 1 for example frequencies relevant for Helfrich et al.'s study). It is understood that this may reflect nested networks that oscillate at different frequencies and spatial scales [4] and that define functional architecture not only by synchronizing at the same frequency but also through complex cross-frequency interactions; this to allow for integration of processes at different temporal and spatial scales [5]–[7]. With respect to the above questions on how the brain operates, the most exciting aspect of oscillatory brain activity is probably that it offers mechanistic accounts. One example is the communication-through-coherence theory [8], which states that the relative timing of oscillatory activity of two neuronal elements enables the control of information transfer, with communication being maximal when phases of high excitability of these elements cycle in synchrony, and minimal when they cycle out of synchrony (see Fig. 1B Model).

**Figure 1:**
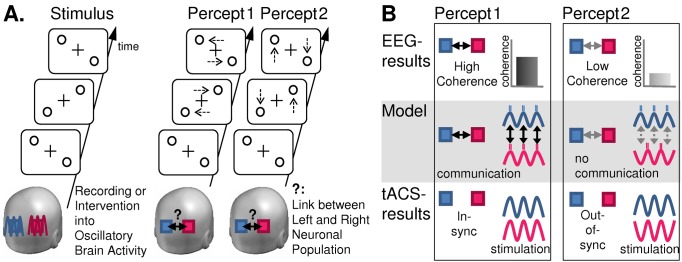
Schematic representation of design, objectives, and insights from the study by Helfrich et al. **A. Design and questions**: Participants viewed an apparent motion stimulus, which elicits a bistable percept consisting of either horizontal (percept 1) or vertical motion (percept 2). A bi-hemispheric network of two posterior areas (blue and red squares) was interrogated as to the functionality of inter-area synchrony (see “?”) in generating these percepts, by recording of brain oscillations through electro-encephalography (EEG), and interventions into these oscillations through transcranial alternating current stimulation (tACS). **B. Results and conclusion**: EEG revealed that the horizontal motion percept was associated with enhanced synchrony (coherence) between oscillatory brain activity of the two posterior areas (as compared to vertical motion percept), in line with coupling of the two areas to a functional network by synchronization of their respective phases of high excitability (see Model). This provides information on a correlative relationship between network activation and function but cannot disentangle whether it is the percept that drives the network, or the network that drives the percept. Intervention with tACS supports the latter. Applying tACS in synchrony over the two areas enhances inter-area coherence of oscillatory activity as well as the horizontal motion percept (as opposed to applying tACS out of synchrony). Hence, synchrony of oscillatory brain activity underlies the formation of functional networks and mediates its associated functions.

**Box 1. Glossary**

**Brain oscillations in the gamma frequency band (gamma-oscillations)**: This is a class of brain oscillations cycling at rapid frequencies (35–100 Hz). Gamma-oscillations are prominent in visual cortex (among other areas) and become evident also in scalp recordings when participants view specific types of visual stimuli.

**Alpha-band brain oscillations** cycle at 8–12 Hz. Alpha-oscillations can co-occur with gamma-oscillations in visual areas, where these two classes of oscillations show an inverse relationship in terms of amplitude.

**Transcranial direct current stimulation (tDCS) and transcranial alternating current stimulation (tACS)** use electrical currents applied through two or more scalp electrodes for transient, non-invasive brain stimulation, whereas **transcranial magnetic stimulation (TMS)** uses the principle of electromagnetic induction. In tACS, the currents are modulated in an oscillatory (sinusoidal) pattern, and can therefore be frequency-tuned to underlying brain oscillations. Likewise, TMS in its rhythmic form (rhythmic TMS) allows for periodic brain stimulation at frequencies of brain oscillations.

The other line of research that is at the heart of Helfrich et al.'s study is concerned with interventions into brain activity by non-invasive brain stimulation techniques; this to probe the brain–behaviour relationship along a more causal dimension [9]. Such techniques are widely used in cognitive and clinical neuroscience, and employ either magnetic or electric fields to stimulate neurons directly (i.e., transcranially) to then test the behavioural consequences. Currently available techniques use transcranial magnetic stimulation (TMS), or a variety of electrical currents such as with transcranial direct current stimulation (tDCS) or transcranial alternating current stimulation (tACS) (see Box 1) [10]. While these techniques have been successfully employed in numerous studies, a recurrent question is how to improve specificity of effects in terms of enhancing focality [11] or targeting specific subpopulations within the stimulated neuronal pool [12]. In addition, simultaneous neuroimaging studies have revealed that the effect of the magnetic or electric field on the stimulated area (under the TMS coil or the stimulation electrode) is spreading to other areas, in many instances along anatomical connections [13],[14]. Hence, any behavioural outcome needs to be interpreted in the context of network effects. Intriguingly, and relevant for interactions with oscillatory brain activity, recent findings indicate that the specificity of these interventions into functionally relevant brain activity may be improved by taking into account not only the spatial dimension (i.e., what anatomical network to stimulate) but also the temporal dimension (what frequency to apply). This is suggested by recent studies using periodic transcranial stimulation protocols (such as tACS or rhythmic TMS) allowing a frequency tuning of stimulation (see Box 1). These studies demonstrate an immediate behavioural effect at specific stimulation frequencies, namely those that match the frequencies of intrinsic brain oscillations[15]–[21]; which may be caused by the periodic stimulation promoting the intrinsic oscillations [22]–[24].

Capitalizing on the above, Helfrich et al. convincingly address in healthy human volunteers the long-standing issue of whether oscillatory brain activity indeed coordinates functional brain architecture, as opposed to representing a mere by-product, and thereby bridge a gap between recordings and interventional studies into brain oscillations (see Fig. 1 for a schematic representation of design, objectives, and insights of the study). They do so by examining the link between visual network activity and specific sensory experiences. To manipulate sensory experience (without changing sensory input), Helfrich et al. employed a visual motion paradigm (see Fig. 1A), in which pairs of diagonally opposed dots are presented on a screen in two alternating configurations (upper left/lower right dots followed by lower left/upper right dots, etc.). This leads to a bistable percept, consisting of time periods during which the two dots are perceived as moving horizontally (see Fig. 1A, apparent motion percept 1), alternating with time periods during which the same dots are perceived as moving vertically (Fig. 1A, apparent motion percept 2). Interestingly, recordings of brain oscillations from left and right occipito-parietal EEG sensors, i.e., from areas processing the right- versus left-sided dots respectively, revealed a temporally stable pattern of relative timing between these oscillations, depending on the percept (replicating [25]): during horizontal motion percepts when the demands for interhemispheric communication can be assumed to be high (as opposed to vertical percepts where motion integration can be resolved within each hemisphere) [26], these left and right oscillations show high coherence in the gamma frequency band (at approximately 35–100 Hz) (Fig. 1B EEG). In other words, oscillations in the left and right occipito-parietal areas are synchronized. This is suggestive of these areas forming a temporally stable network during horizontal as opposed to vertical motion integration, in line with models of network coordination by synchronization of brain oscillations (Fig. 1B Model) [8],[27]. Importantly, applying rhythmic brain stimulation in synchrony over the left and right occipito-parietal cortex using tACS at gamma frequency enhances both the gamma-band EEG coherence between the two hemispheres (without affecting gamma-power) and its associated percept (i.e., horizontal motion), as opposed to applying gamma-tACS out of synchrony (Fig. 1B tACS). See also Polania et al. [19] for a conceptually similar tACS result, without the direct evidence for concurrently enhanced EEG synchrony. This shows that in-synchrony tACS versus out-of-synchrony tACS over two elements of an oscillatory visual network can be used to stabilize/destabilize this network, and with meaningful perceptual consequences. This is in accord with brain oscillations not only indexing network coordination and associated functions, but causing them.

The findings of Helfrich et al. make an important contribution. They more firmly link the dynamics of oscillatory brain activity to the formation of functional networks, as well as the orchestration of brain function (here phenomenological experience) and this along a causal dimension. This corroborates and extends a growing number of studies showing that brain oscillations can serve as targets for controlled interventions into brain activity and function, by non-invasive brain stimulation in periodic patterns [22]–[24]. The principle idea is to promote brain oscillations that have been associated with specific functions (as inferred from correlative brain-behavioural links) to cause performance changes, provided a causal relationship underlies the correlative data. For instance, it has been shown that promoting oscillations of the parietal cortex known to be related to attentional selection using frequency-tuned rhythmic TMS [22] biases perception towards the expected stimulus dimension [17],[20]. Likewise, tACS (or oscillatory tDCS) tuned to fronto-temporal oscillations, which have been associated with memory consolidation during slow-wave sleep or dream patterns during REM-sleep (e.g., lucid dreaming), have been shown to enhance memory or lucid dream content, respectively [15],[21]. And equivalent effects have been found for oscillatory motor system activity [16],[18]. This opens powerful opportunities for neuroscience and clinical interventions, not only allowing to test models of how brain activity implements function but also how it relates to dysfunction, to inform controlled intervention into the brain–behaviour relationship.

These findings are exciting and indicate that it is promising to study brain oscillations, even at a macroscopic scale (such as measured with EEG/MEG), to answer some of the long-standing questions of how the brain works. They also take the emerging new approach of using periodic transcranial stimulation to interact with brain oscillations and function beyond the proof-of-principle stage. However, the usefulness of this approach will depend on the extent to which its specificity can be improved (e.g., up- versus down-regulating oscillations, tailoring to individual differences) and its mechanisms of actions understood. One unresolved point is the spatial extent of stimulation. With tACS, the conventional stimulation electrodes are large (several cm^2^) and require a “return” electrode which excites widespread areas. To render stimulation more focal, special electrode montages have been proposed [11], as also used by Helfrich et al., and which may explain some of the differences to a previous study of the same group using a less focal electrode montage [28]. Other developments are underway to funnel stimulation to specific target areas by the use of multichannel electrode configurations and computational (forward) models of electrical field distributions [29]. In this context, it will be of interest to compare the efficiency of frequency-tuned tACS with frequency-tuned rhythmic TMS, the latter thought to be more focal, but also more superficial. In addition, it is still largely unknown how these forms of rhythmic stimulation interact with intrinsic brain oscillations. There is growing evidence that the periodic electric or magnetic force may entrain the underlying oscillations during stimulation [22],[23], and that long-lasting effects may arise from this entrainment, possibly by inducing plasticity effects via spike-timing dependent plasticity in the circuits generating these oscillations [30]. It is the former, short-term effects that are of interest for experimental interventions in cognitive neuroscience for testing theory (because of their limited duration), but the latter, longer-lasting effects that are of relevance for clinical interventions. Finally, while Helfrich et al. report cross-frequency effects of gamma-tACS, in particular in the alpha frequency band (8–12 Hz), it remains to be studied in detail how the induced oscillations resonate in other, nested oscillatory networks. These and other points will need to be resolved in future work to be able to fully assess the extent of the impact of this emerging approach.

## Abbreviations

EEG/MEG: electroencephalography/magnetoencephalography
tACS: transcranial alternating current stimulation
tDCS: transcranial direct current stimulation
TMS: transcranial magnetic stimulation
